# A review of transformer models in drug discovery and beyond

**DOI:** 10.1016/j.jpha.2024.101081

**Published:** 2024-08-30

**Authors:** Jian Jiang, Long Chen, Lu Ke, Bozheng Dou, Chunhuan Zhang, Hongsong Feng, Yueying Zhu, Huahai Qiu, Bengong Zhang, Guo-Wei Wei

**Affiliations:** a Research Center of Nonlinear Science, School of Mathematical and Physical Sciences, Wuhan Textile University, Wuhan, 430200, China; b Department of Mathematics, Michigan State University, East Lansing, MI, 48824, USA; c Department of Electrical and Computer Engineering, Michigan State University, East Lansing, MI, 48824, USA; d Department of Biochemistry and Molecular Biology, Michigan State University, East Lansing, MI, 48824, USA

**Keywords:** Transformer, Drug discovery, Chemical language understanding, Molecular dynamics, Protein design

## Abstract

Transformer models have emerged as pivotal tools within the realm of drug discovery, distinguished by their unique architectural features and exceptional performance in managing intricate data landscapes. Leveraging the innate capabilities of transformer architectures to comprehend intricate hierarchical dependencies inherent in sequential data, these models showcase remarkable efficacy across various tasks, including new drug design and drug target identification. The adaptability of pre-trained transformer-based models renders them indispensable assets for driving data-centric advancements in drug discovery, chemistry, and biology, furnishing a robust framework that expedites innovation and discovery within these domains. Beyond their technical prowess, the success of transformer-based models in drug discovery, chemistry, and biology extends to their interdisciplinary potential, seamlessly combining biological, physical, chemical, and pharmacological insights to bridge gaps across diverse disciplines. This integrative approach not only enhances the depth and breadth of research endeavors but also fosters synergistic collaborations and exchange of ideas among disparate fields. In our review, we elucidate the myriad applications of transformers in drug discovery, as well as chemistry and biology, spanning from protein design and protein engineering, to molecular dynamics (MD), drug target identification, transformer-enabled drug virtual screening (VS), drug lead optimization, drug addiction, small data set challenges, chemical and biological image analysis, chemical language understanding, and single cell data. Finally, we conclude the survey by deliberating on promising trends in transformer models within the context of drug discovery and other sciences.

## Highlights

• This paper reviews the diverse applications of transformers in drug discovery and beyond.
• The review delineates the advantages and weakness of various transformer architectures.
• The review discusses the promising trends of transformer models in drug discovery and beyond.

## Graphical abstract

**Figure:**
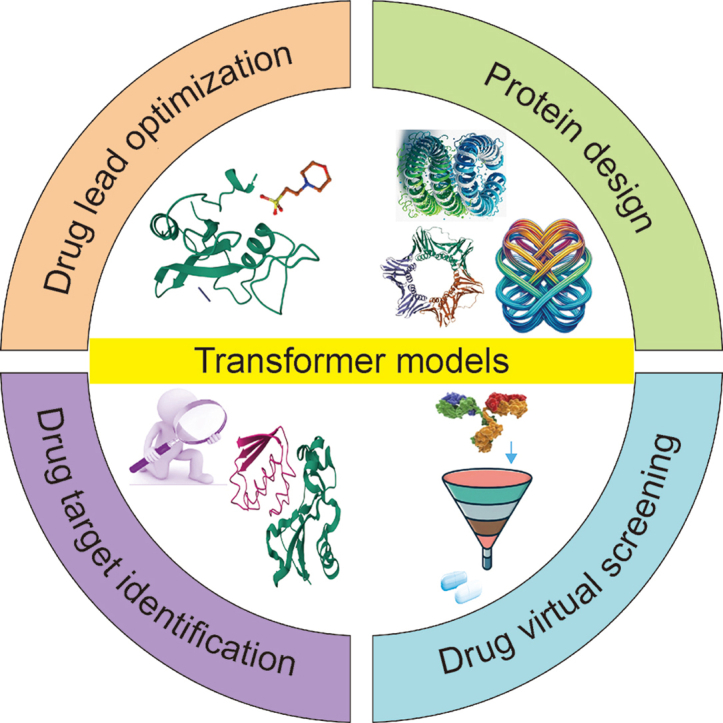

## 1 Introduction

In 2014, Bahdanau et al. [1] introduced the attention mechanism to natural language processing (NLP) models, laying the groundwork for transformer models [2]. Initially designed for automatic translation tasks in NLP, transformers have proven to be highly adaptable and flexible, finding applications across a wide array of fields beyond NLP. Through suitable modifications and adjustments, transformers have been successfully applied in computer vision (CV) [3], image processing [4], chemistry [5], and life sciences [6]. Since their introduction in 2017, transformers have garnered widespread attention due to their versatility, leading to the continual emergence of various transformer-based models and applications, including generative pre-trained transformers (GPT) [7] and bidirectional encoder representations from transformers (BERT) [8], among others. Even in prominent artificial intelligence (AI) applications such as large language models (LLMs) and chatbots, like ChatGPT [9], and others, transformer-based models remain a central component, paving the way for a deeper comprehension of molecular structures, sequences, and functionalities [10,11].

In the past few years, there has been a growing fascination with harnessing deep learning (DL) technology, notably the rise of transformer models, to reshape methodologies within drug design and discovery. Several factors contribute to the notable achievements of transformer models in these domains.

Firstly, the inherent capability of transformers to handle long-range dependencies plays a pivotal role in processing intricate molecular structures and chemical reactions. In contrast to conventional recurrent neural networks (RNNs), which grapple with capturing long-term dependencies in sequential data, transformers excel in discerning complex relationships among atoms, molecules, and materials across extensive distances. This proficiency enables transformer models to make accurate predictions of drug properties, decode drug molecular structures, and visual screening by comprehensively considering the interplay of myriad factors across diverse spatial scales. Consequently, transformer-based models offer a more comprehensive and nuanced understanding of chemical systems, thereby enhancing the precision and dependability of predictions in drug discovery.

Moreover, the self-attention mechanism integrated into transformer architectures enables efficient parallel processing, a critical advantage in managing the extensive data sets inherent in the investigations of drug discovery. Traditional RNNs handle sequential data sequentially, resulting in computational inefficiencies, particularly when confronted with sizable data sets or intricate molecular arrangements. In contrast, transformers possess the capability to simultaneously attend to multiple positions within a sequence, facilitating parallel computation of features and interactions across diverse regions of drug molecules. This parallelization expedites both model training and inference, streamlining experimentation and analysis in the realms of drug discovery. Consequently, transformer-based models expedite the elucidation of drug physical and chemical properties, drug molecular behaviors, and drug chemical processes, thereby accelerating research advancements in these domains.

Furthermore, the scalability and adaptability inherent in transformer architectures render them highly suitable for tackling a myriad of challenges in drug discovery. Transformers can be easily customized and fine-tuned to accommodate diverse data types, ranging from molecular representations to experimental measurements, facilitating seamless integration with existing research methodologies and data sets. Additionally, transformer-based models demonstrate robust performance across a broad spectrum of tasks, spanning from predicting drug properties to designing novel drug molecules, thanks to their flexible architecture and extensive pre-training capabilities. This versatility empowers researchers to apply transformer models to a plethora of problems in drug design and discovery with promising outcomes. Hence, the scalability and adaptability of transformer architectures significantly contribute to their widespread adoption and success in advancing the frontiers of research in the drug discovery domain.

Overall, the transformer architecture has been widely applied to diverse aspects in drug discovery. Noteworthy applications within this framework include drug target identification [12,13], transformer-enabled drug virtual screening (VS) [14,15], drug lead optimization [16,17], drug addiction [18,19], protein design and protein engineering [20,21], small data set challenges [22,23], molecular dynamics (MD) [24], chemical and biological image analysis [25,26], and chemical language understanding [27,28]. Despite their prevalence, there is a lack of an organized taxonomy linking these aspects in existing literature. There are a few existing review articles about transformers, but most of them centralized the application domain in computer science [3], medicine [29,30], vision [31], text [[32], [33], [34]], and others [35]. None of them focus on the problems in drug discovery. Therefore, there is a need to provide a research-area-centered review of the status so that it can benefit readers interested in drug discovery.

The rest of this paper is organized as follows. Section 2 offers a review of basic transformer technology. In Section 3, various primary applications of transformer-based models in drug discovery are discussed, encompassing drug target identification, transformer-enabled drug VS, drug lead optimization, drug addiction, protein design and protein engineering, small data set challenges, MD, chemical and biological image analysis, chemical language understanding, and single cell data. Lastly, Section 4 presents perspectives on future developments, including the role of Chatbots and Sora in drug discovery, multimodal learning for omics data, catalyst and enzyme screening, and customizing transformer models for drug discovery.

## 2 Technical essence of transformers

Language modeling has made impressive progress in the past few years, largely owing to the invention of the transformer architecture, which has sparked a revolution in many fields of machine learning (ML) and made breakthroughs in chemistry and material science.

**Fig. 1:**
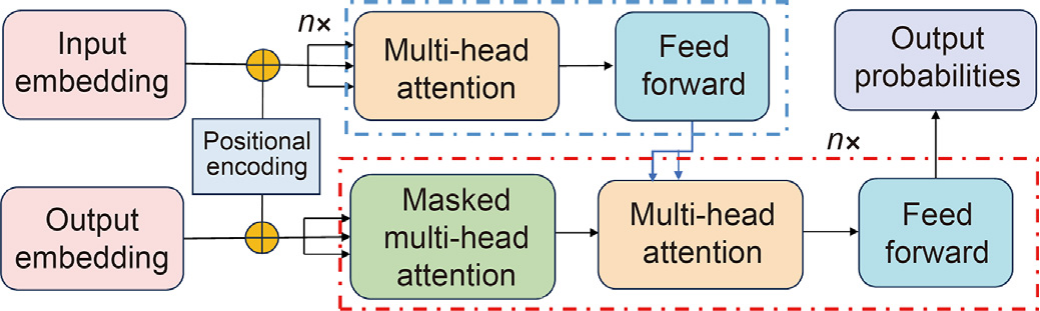
Schematic diagram of transformer model. The transformer model consists of an encoder, decoder, self-attention mechanism, and feedforward neural networks.

The transformer model, proposed by Vaswani et al. [2], is the first sequence transduction model entirely based on the attention mechanism, comprising two main components: the encoder and the decoder. Prior to this, most sequence transduction models relied on complex RNNs or convolutional neural networks (CNNs). The transformer model adopts a novel structure that encodes input data into features with strong representation ability through the attention mechanism [36], replacing the loop layer in the traditional encoder-decoder architecture with the multi-head attention mechanism [2]. A deep understanding of the structural framework and working principle of the transformer model can aid in comprehending its application in the fields of chemistry and materials science. The illustration of the framework of the transformer is provided in Fig. 1.

In the transformer model, the encoder plays a pivotal role in transforming the input sequence, regardless of its length, into a fixed-dimensional vector representation. This process is critical as it provides compression and an abstract representation of the sequence for subsequent decoders. The decoder then converts this fixed-length vector back into a variable-length sequence of the target language.

The encoder comprises multiple structurally consistent layers known as encoder layers. Within each encoder layer, several core components synergize. 1) A multi-head self-attention mechanism enables the model to focus on elements at different positions during the processing of input sequences, thereby enhancing the model's comprehension of the internal relationships within the sequence [37]. 2) Feedforward neural networks provide the model with the capability to execute nonlinear transformations, facilitating the acquisition of more intricate feature representations. 3) Residual connections [38] and layer normalization mitigate the issue of vanishing and exploding gradients during deep network training, enabling the model to learn more robustly and effectively. These components collaborate harmoniously to empower the encoder in processing sequence data effectively and generating high-quality vector representations. The transformative aspect of the transformer model, relative to preceding models, lies in the incorporation of the multi-head self-attention mechanism in encoders [37].

The self-attention mechanism empowers the model to dynamically distribute attention across various segments of the input data during processing. Consequently, the model can prioritize information crucial to the current task, a process known as “attention concentration”. Through attention concentration, the model selectively focuses on key segments while reducing attention on others. At the heart of the self-attention mechanism lies the model's capacity to leverage contextual information from the entire input sequence, rather than relying solely on specific positions or elements, to construct the representation of each element. This holistic approach enables the model to make decisions based on the overall information of the input sequence. The computational cost of transformers is heavily influenced by the length of the sequence data due to the self-attention mechanism, which requires each token in the sequence to attend to every other token. This results in a quadratic scaling of computational and memory costs, represented as *O* (*n*^2^) where *n* is the sequence length. Consequently, as the sequence length increases, the resources needed to process the data grow rapidly, posing challenges for handling very long sequences efficiently.

The multi-head attention mechanism enables the model to utilize multiple independent attention heads in parallel to capture distinct representation subspaces of the input sequence. Each attention head calculates a weighted sum of the input sequence using learned weights. With unique weight parameters for each head, the model can simultaneously attend to different aspects of the input sequence. Subsequently, the outputs of the multi-head mechanism are concatenated and subjected to a linear transformation before being passed to the next layer of the network.

The advantage of the multi-head attention mechanism lies in its ability to collectively attend to information from various representation subspaces at different positions, thereby enhancing the model's representation capability. Each attention head can specialize in specific aspects of the input sequence, facilitating the model's capture of complex data interactions. Moreover, multi-head attention achieves parallelized computation, with each head capable of independent calculations, thus significantly improving computational efficiency and enabling the model to handle larger data sets.

## 3 Applications to drug discovery and beyond

### 3.1 Protein design and protein engineering

Proteins are crucial for numerous physiological functions in the human body, driving essential life processes. Protein engineering aims to design or discover proteins with specific functions, such as enhancing phenotypes, improving enzyme catalysis, increasing resistance to climate changes, and boosting antibody efficacy [39]. Since its inception in the 1950s, protein engineering has advanced significantly with DNA sequencing and polymerase chain reaction (PCR) technologies. Traditional methods, including directed evolution [40] and rational design [41], are limited by the need for extensive experimental screening across a large mutation space, resulting insignificant time and cost constraints [42].

**Fig. 2:**
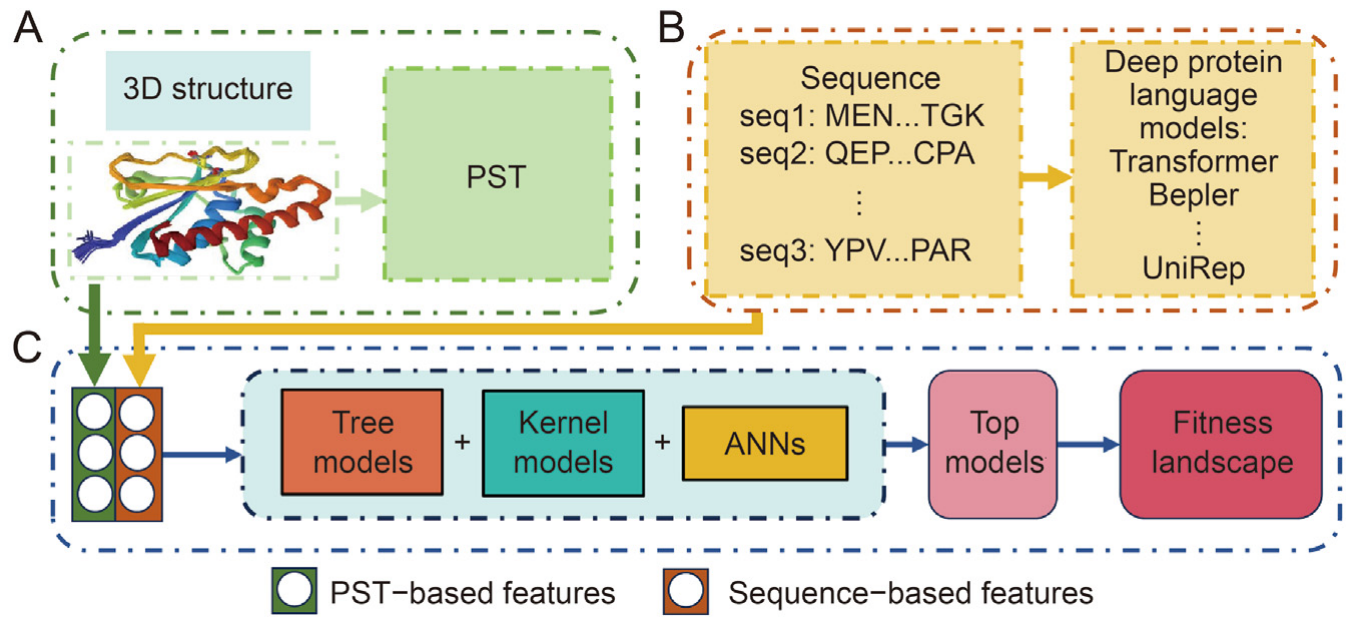
Conceptual diagram of Topology-offered protein Fitness (TopFit). (A) Obtain protein three-dimension (3D) structure from Protein Data Bank (PDB) database and generate persistent spectral theory (PST) embedding. (B) Generate sequence-based embedding and evolution scores from deep protein language models. (C) Connect two different features and select the optimal model from the three types of models (tree models, Kernel models, and artificial neural networks (ANNs)) to obtain a fitness landscape.

In recent years, the advent of data-driven ML models, particularly transformer-based models such as Topology-offered protein Fitness (TopFit) [20] and the multiple sequence alignment (MSA) transformer [43], has significantly reduced the cost and accelerated the process of protein engineering. These models are known for their exceptional adaptability and performance. TopFit, a topology-based ML model, can create a protein-to-fitness map (i.e., fitness landscape) from sparsely sampled experimental data, as illustrated in Fig. 2. It outperformed sequence-based embeddings on almost all 34 data sets with a persistent spectral theory embedding. Meanwhile, the MSA transformer is effective in predicting mutation effects.

Transformer-based models, such as AlphaFold2 [44], AlphaFold3 [45], and EnsemPPIS [46], accurately predict protein structures, aiding in understanding protein functions and protein design. Transformer variants like PeptideBERT [47] can predict protein properties, such as solubility and hemolysis, which are essential for protein design. These models enable the simulation and modification of protein sequences, facilitating novel protein design with significant implications for disease prevention, treatment, and drug development.

Pre-trained deep transformers have had tremendous success in a wide variety of molecular fields. However, essentially all transformers in molecular science are built upon molecular sequences, such as DNA, RNA, protein amino acid, and simplified molecular-input line-entry system (SMILES) sequences. As a result, vital stereochemical information, which is essential for various molecular functions, is neglected. Wee et al. [48] integrated persistent Laplacians and pre-trained transformer for protein solubility changes upon mutation. Qiu and Wei [20] utilized a pre-trained transformer in persistent spectral theory-guided protein engineering. In these approaches, algebraic topology-based structure embedding complements protein sequence embedding obtained from pre-trained transformers and plays an essential role in these studies. The essence is that sequence-based transformers may not capture all the stereochemistry in bioinformatics. Advanced topological data analysis (TDA) approaches, such as persistent Laplacians [49], persistent path Laplacians [50], and persistent hyper digraph Laplacians [51], are designed to extract stereochemical information in molecules and play an important role in drug discovery.

Transformer-based models have fundamentally transformed the prediction of protein properties, such as ligand binding affinity and antibody efficacy, by skillfully deciphering the intricate relationship between protein sequence and structure. At the heart of the transformative impact of transformer models lies their capacity to discern subtle patterns within protein sequences, shedding light on crucial properties such as secondary structure, solvent accessibility, ligand binding sites, and protein-protein interactions with remarkable precision. This not only enriches our theoretical comprehension of proteins, but also drives progress in drug discovery.

Although transformer models have made progress in protein engineering, they still face some challenges. For example, accurate prediction of large multi-domain proteins with complex topological structures [21] and accurate scoring of protein binding interfaces [52] require some improvements and optimizations for the transformer models to effectively process these complex biological data. Overall, the application of transformer models in the fields of protein design and protein engineering provides scientists with powerful tools, which are expected to greatly promote the development of drug discovery and make important contributions to human health and well-being.

### 3.2 MD

MD, which began in the early 1950s to study interactions among hard spheres, has evolved into a vital tool in drug discovery. MD uses computer simulations to model molecules by iteratively calculating atomic and molecular interactions and their spatial motions. This allows for detailed observation of dynamic phenomena over time, providing deep insights into molecular behavior.

The self-attention mechanism in transformer models enhances the efficiency and accuracy of processing complex molecular data, revealing unprecedented insights into molecular structure, kinetics, and interactions [53]. Integrating transformer models with MD is expected to offer innovative tools and perspectives, significantly advancing the field of drug discovery.

In recent years, DL-based MD modeling (DLMD) has gained prominence across various domains [54]. Wu and Li [55] recently introduced the diffusion model for MD simulations (DiffMD), which achieves more accurate modeling of molecular systems by directly estimating the gradient of the logarithmic density of molecular conformations. Specifically, DiffMD achieved the lowest accumulative root mean square error (ARMSE) on all eight organic molecules and exceeded all baselines by a large margin for the five C_7_O_2_H_10_ isomers with the largest ARMSEs in the data set.

Peptides hold significant research potential in drug discovery, with MD offering efficient means to gather large-scale peptide data sets. Nonetheless, enhancing prediction accuracy remains a pressing concern. Liu et al. [24] initially aggregated a high-quality and large peptide self-assembly simulation data set comprising 62,159 peptide samples generated by coarse-grained MD (CGMD) in 2023. Subsequently, they employed state-of-the-art models based on amino acid sequence coding, encompassing RNN, long short-term memory (LSTM), and transformer sequence models, as well as structural DL models. Extensive benchmarking studies revealed that the transformer, as a DL model based on sequence encoding, outperformed the other four and nine benchmark models on regression and classification tasks, respectively.

The integration of transformer models with MD techniques offers a powerful approach for drug discovery by enhancing the analysis and manipulation of molecules. MD provides several advantages, including complete control over all parameters, allowing for diverse system interventions and detailed characterization of structural evolution. It is extensively used in biology to model the structural and dynamic behaviors of biomolecules such as proteins and nucleic acids.

Despite these advantages, MD faces limitations due to restricted computational resources, which hinder long-term process simulations. Additionally, the reliability of MD results depends on the quality of the models used and often requires experimental validation. However, ongoing methodological advancements and technological progress promise that the combination of transformer models and MD will provide robust support in tackling scientific challenges related to complex molecular systems. This fusion is set to open new possibilities in drug discovery, as well as in biophysics, chemistry, and materials science.

### 3.3 Drug target identification

In recent years, with the rapid development of the field of bioinformatics, the superior performance of transformer models has gradually been recognized and applied in drug target identification tasks. Drug targets refer to biomolecules associated with diseases or pathological states, and their identification is the foundation of drug development [56].

Traditional drug target identification is mainly carried out through biological experiments or high-throughput screening (HTS) techniques, which often require a lot of time and money costs, so the number of drug molecules that can be screened is very limited. With the rapid development of science and technology, various DL and ML models, such as transformers, have emerged one after another, providing a new way for drug target identification. This greatly saves the cost of drug target identification and accelerates the process of drug research and development. For example, the network-based DL methodology (deepDTnet) can recognize molecular targets in known drugs on a computer, accelerate drug reuse, and minimize conversion gaps in drug development [12].

The sequence-based transformer protein language model (QuoteTarget) is an innovative tool for identifying potential drug targets. It can independently extract features from amino acid sequences for drug target protein identification, which has potential significance for identifying drug binding sites [13], because it does not rely on traditional biological experimental methods, but only extracts effective information from sequence data. This not only improves the efficiency of drug target identification, but also may discover new drug targets that traditional methods cannot discover. Additionally, Chen et al. [57] proposed the concept of sequence-to-drug in 2023, which is based on a computationally designed drug with end-to-end differentiable protein sequence information. Its core tool is TransformerCPI 2.0, which not only has the ability to generalize across proteins and compounds and to discover new challenging drug targets, but also identifies new targets for existing drugs through the reverse application of the concept. The emergence of this model provides a powerful tool for drug developers, greatly improving the efficiency of drug target identification. Furthermore, the biomedical generative pre-trained based transformer language model BioGPT can be used to identify potential targets related to aging [58], further expanding the application of transformer models in the field of drug design. The majority of these applications have been limited by their dependence on biological sequences, which overlook crucial three-dimensional (3D) structural information that is incompatible with the sequential nature of NLP models.

**Fig. 3:**
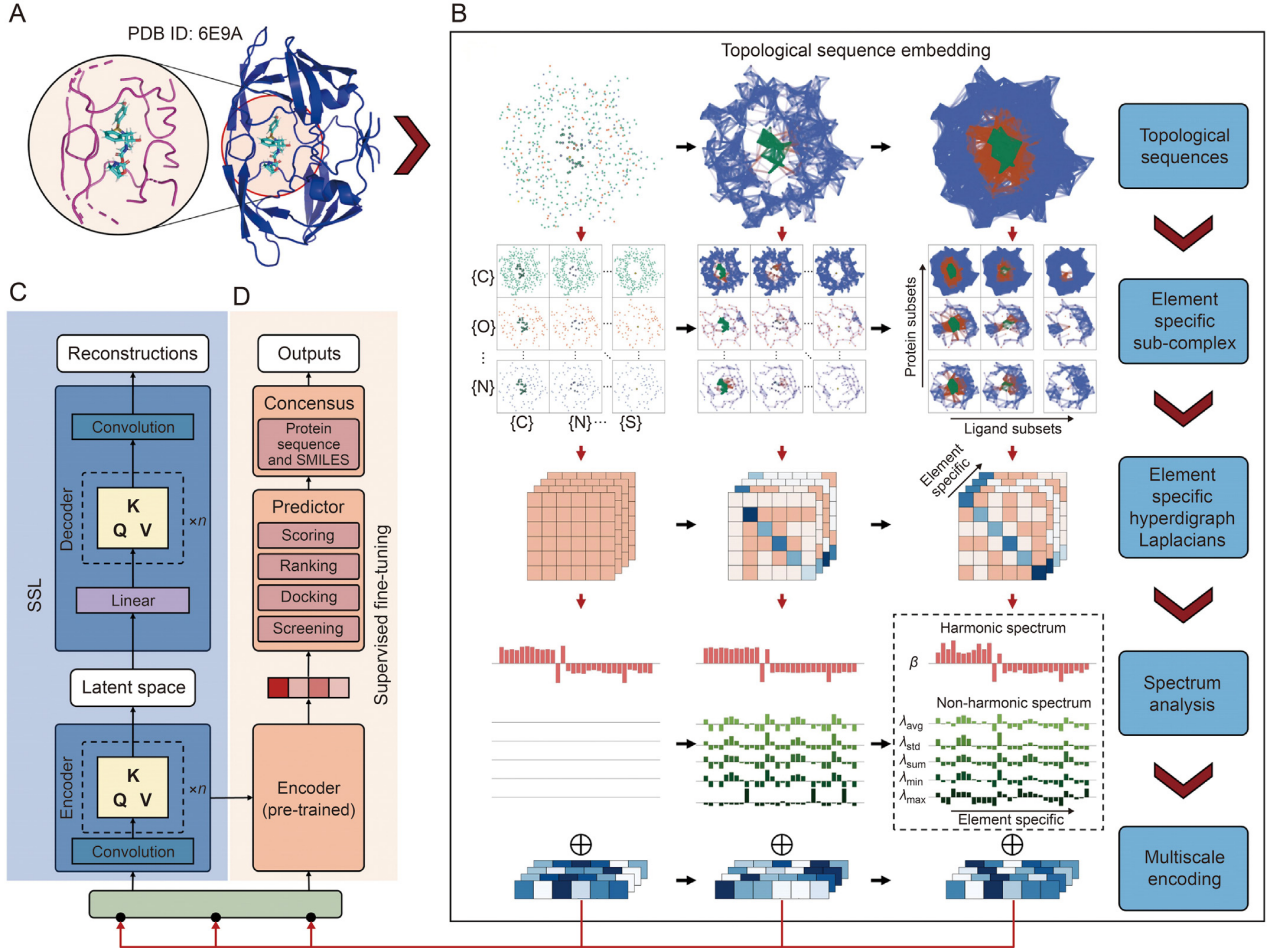
A framework diagram of the overall topological transformer (TopoFormer) model. (A) A three-dimension (3D) protein-ligand complex (Protein Data Bank (PDB) ID: 6E9A) and its interactive domain. (B) The topological sequence embedding of a 3D protein-ligand complex. From top to bottom, there are five steps during the process of embedding, including topological sequences, element specific sub-complex, element specific hyperdigraph Laplacians, spectrum analysis, and multiscale encoding. (C) Self-supervised learning (SSL) is used to unlabeled topological sequences for both transformer encoder and transformer decoders. (D) Task-specific protein-ligand complex data are fed into the pre-trained encoder during the process of supervised fine-tuning. C: carbon; O: oxygen; N: nitrogen; S: sulfur; avg: average; std: standard; K: key; Q: query; V: value; SMILES: simplified molecular-input line-entry system.

Recently, a new transformer, TopoFormer, built entirely on 3D molecular structures, has been proposed to capture essential interactoins across spatial scales [59]. The essential idea is to convert 3D structures into topological sequences admissible to NLP models, including transformers, by using multiscale topological Laplacians [49,51,60] as depicted in Fig. 3. Consequently, the TopoFormer-based model outperformed others across three benchmark data sets for scoring tasks. It achieved success rates of 72% for low-level measurement and 63% for high-level measurement on CASF-2007 for ranking tasks. For docking tasks, it achieved success rates of 93.3% on CASF-2007 and 91.3% on CASF-2013 [59].

Although transformer models have shown great potential in the field of drug design, such as predicting drug target interactions [61], drug target affinity [62], drug reactions [63], and designing antiviral drug molecules [64], etc., they are still in the initial stage of drug target recognition tasks and there are still many challenges to overcome in the future. The most significant aspects are the interpretability and generalization ability of the model. In addition, the limited drug target data is a key issue. Although there is a lot of known drug and target information, the drugs that have been experimentally validated and approved are very limited. Therefore, the drug target data available for model training and learning is very limited, which may lead to the model not being able to learn enough features, thereby affecting the accuracy of model identification. Nevertheless, with the continuous development of the field of bioinformatics and the continuous optimization of these models, we believe that the transformer model can overcome the above challenges and achieve more significant results in drug target identification tasks.

### 3.4 Transformer-enabled drug VS

VS is a powerful computational technique used in drug discovery to quickly identify potential drug candidates from large libraries of chemical compounds. It plays a crucial role in the early stages of drug development by helping researchers prioritize the most promising compounds for further experimental evaluation [[65], [66], [67]]. By using computer algorithms and molecular modeling techniques to predict the likelihood of compounds binding to a target protein [68], VS offers a cost-effective and efficient alternative to traditional methods. This technique simulates interactions between small molecules and target proteins in silico, allowing researchers to rapidly narrow down potential drug candidates, saving both time and resources. VS is applied in various stages of drug discovery, including hit identification, lead optimization [14], and drug repurposing [15].

Recently, a new approach has emerged in VS that utilizes sequence information from both protein and ligand without resorting to any 3D structures [69]. This approach depends on advanced NLP models, such as autoencoders or transformers. Transformer-enabled sequence-based VS (SVS) models have significantly improved efficiency of traditional VS. Shen et al. [69] developed a transformer-enabled sequence-based virtual screen (SVS) platform. The essence is to utilize transformer representations for both protein and ligand and thus bypass 3D structure-based docking process. The protein representations were obtained from the pre-training of 250 million sequences [6], while the ligand representations were attained from the pre-training of over 700 million small molecules [10,70].

**Fig. 4:**
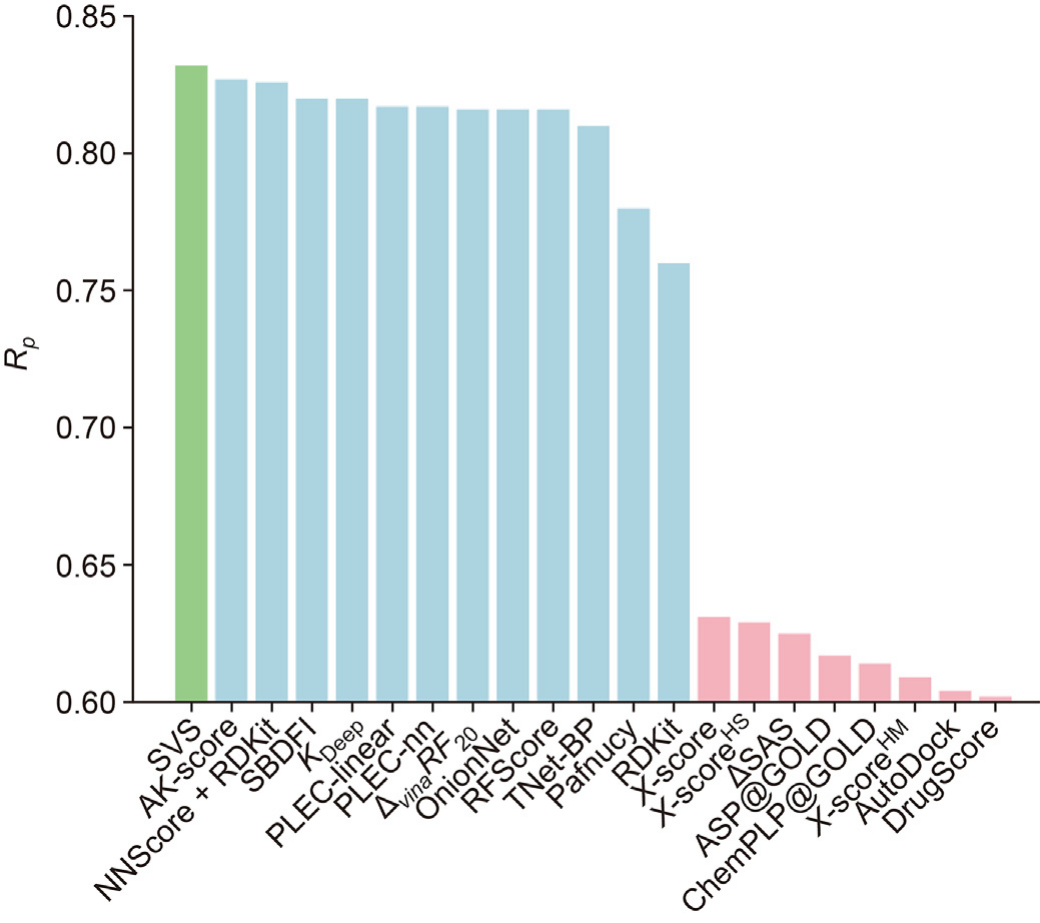
Comparison of the Pearson's correlation coefficient (*R*_*p*_) of the transformer-enabled sequence-based virtual screening (SVS) model and those of other structure-based approaches for the protein-ligand binding affinity prediction of the PDBbind-2016 core set. Results in lime, ocean, and rose colors are obtained using SVS, experimental structures, and docking generated structures of protein-ligand complexes, respectively. Transformer-enabled SVS outperforms the state-of-the-art models [69]. Reprint from Ref. [69] with permission.

Fig. 4 [69] shows the comparison of Pearson's correlation coefficient (*R*_*p*_) of the transformer-enabled SVS model (lime) and those of other structure-based approaches for the protein-ligand binding affinity prediction of the PDBbind-2016 core set. The PDBbind-2016 core set is a benchmark data set in drug design and discovery [71]. It has been widely used to examine various methods in the literature [68]. As shown in Fig. 4, the transformer-enabled SVS model outperforms other methods in the field, which is a remarkable achievement. Transformer-enabled SVS has the potential to transform current practices in drug discovery, enzyme design, and protein engineering.

Predicting the active binding of drugs to target proteins is seen as a central challenge in VS [72]. Liu et al. [73] proposed a new model called the intermolecular graph transformer (IGT) in 2022. This technique employed a specially designed attention mechanism to successfully and efficiently model the correlation information between molecules with the help of a transformer-based three-channel architecture. IGT significantly outperformed current state-of-the-art methods in terms of active binding and binding pose prediction by 9.1% and 20.5%, respectively. Encouragingly, IGT showed good adaptability to unknown receptor proteins. This work highlighted its potential application in drug screening in the face of severe acute respiratory syndrome coronavirus 2 (SARS-CoV-2).

**Fig. 5:**
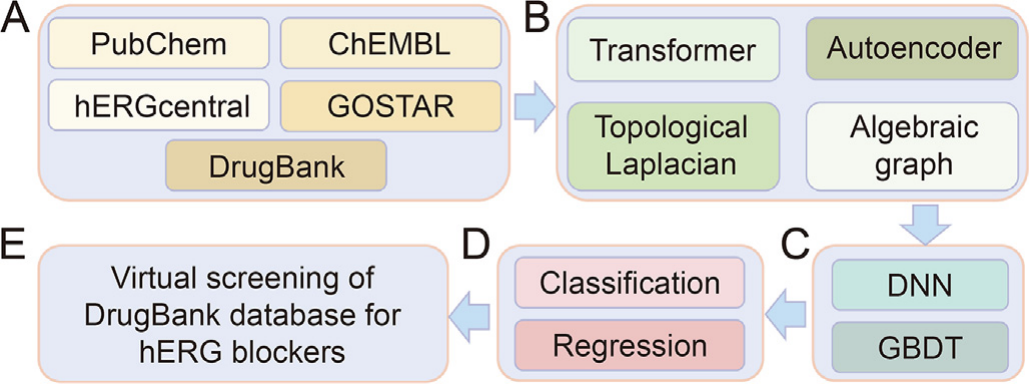
Schematic for creating the machine learning (ML) platform. This platform uses trained classification and regression models to perform virtual screening (VS) of the human ether-à-go-go-related gene (hERG) blockers in the DrugBank database. (A) Classification and regression models are based on data sets from the GOSTAR, hERGcentral, PubChem, and ChEMBL databases. Compounds in the DrugBank database are screened for hERG blocking. (B) Based on NLP, transformers and autoencoders provide sequence information. Topological Laplacians and algebraic graphs provide complementary three-dimension (3D) structure information based on advanced math. (C) Combining the information from molecular embeddings to create machine learning models using gradient boosting decision tree (GBDT) and deep neural network (DNN) algorithms. (D) Training classification and regression models. (E) A VS operation with trained classification and regression models for hERG blockers in the DrugBank database [74]. Reprint from Ref. [74] with permission.

In the early drug discovery phase, a comprehensive assessment of the human ether-à-go-go-related gene (hERG) blocking activity appears to be crucial. The latest study was done by Feng and Wei [74] in 2023, who constructed an innovative ML model for VS of potential hERG blockers in the DrugBank database. The flowchart of this model is shown in Fig. 5 [74]. In this study, two NLP methods, namely autoencoders and transformers, were skillfully applied to the embedding of molecular sequences. Among them, transformer refers to the bidirectional encoder transformer (BET) model, focusing on the encoder architecture. To more fully consider the 3D structure of molecules, the team also introduced topological Laplace operators and algebraic graphs. They found that 227 of the 8,641 compounds in DrugBank could be potential hERG blockers, an innovative finding that provided a strong guide for future experimental studies.

VS technologies are increasingly vital in the discovery of lead compounds [75]. Whether used alongside HTS or independently, VS provides a rapid and cost-effective method for identifying novel bioactives [76]. The effectiveness of VS depends on the quantity and quality of available data and the predictability of the underlying algorithms [77]. Transformer-based models are particularly effective in this domain, offering significant advantages for drug design [78].

Transformers excel in parallel processing, which is beneficial for handling large data sets and extracting meaningful features [79]. Additionally, they can infer extensive information, including evolutionary history, 3D structures, and biochemical properties [6]. The successful application of transformer models in VS tasks for chemical molecules and biomolecular domains underscores the potential of DL to enhance drug design significantly.

### 3.5 Drug lead optimization

The optimization of drug lead compounds is an important step in the drug discovery process, which refers to the structural changes made to the pre-screened drug molecules to improve their properties. This process is crucial because only molecules that possess multiple properties such as safety, drug producibility, stability, and large-scale preparation are likely to become drugs suitable for the human body and ultimately be marketed.

With the proposal and rapid development of DL algorithms, the development of AI has also ushered in a boom. DL models have become active in various fields with their own advantages, providing an efficient tool and method for biology, chemistry, and materials science. In the field of drug lead optimization, the application of DL models is changing the research and development process of this field at an unprecedented speed. For example, an AI method for lead compound optimization proposed in 2023, pairwise binding comparison network (PBCNet), can accelerate lead optimization projects by an average of 473% [16]. This method significantly improves the speed of drug development. In addition, Mol-CycleGANl based on the CycleGAN model can generate optimized compounds with high structural similarity to the original compound [17]. This technology provides a new perspective and powerful tools for drug design.

The purpose of drug lead optimization is to improve the drug properties of molecules. Traditional drug lead optimization relies on experimental methods and the intuition and experience of chemists [80], involving complex multi-step experiments, which not only consume a long time but also have low efficiency. In this context, the field of drug lead optimization requires a new, low-cost, and easy to implement method. Thanks to the introduction of the transformer model, new solutions have been provided for this problem. For example, the constraints transformer (C-Transformer) model can retain the core scaffold of the original molecule after training, while selectively modifying the original molecule as needed, and the modified molecule still retains biological activity against biological targets. This not only provides scientists with a reliable molecular optimization tool, but also ensures both effective pharmacodynamics (PD) and safe pharmacokinetics (PK) [81]. In addition, the Transformer-R model proposed by He et al. [80] in 2021 can generate R-groups and attach them to the core of the original molecule while determining the initial molecule and specified properties, thereby creating new molecules that meet specific needs. In addition, the transformer model can generate new molecules with specific properties by accurately optimizing the initial molecules, providing a powerful molecular optimization tool for drug development [82].

Although transformer models are widely used in the field of drug lead optimization, they also face some challenges. Firstly, there are limitations on data resources. The training of transformer models often relies on large-scale data sets, but the construction of these data sets often requires a lot of time, and the variety and complexity of drug molecule data may affect the generalization ability of the model. Secondly, the validation of optimized drug molecules is also a problem. The results output by the transformer model often has predictive properties, requiring experimental verification and screening in the optimized stage, which undoubtedly increases the cost and workload of research and development. Finally, the adaptability of the model is also a key issue. Drug design is a constantly changing field, and the discovery of new targets and the emergence of new compounds all pose new requirements for models. Therefore, how to make the model quickly adapt to new transformations is still a question worth considering.

### 3.6 Drug addiction

Drug addiction refers to a chronic relapsing brain disease characterized by compulsive drug use and recurrent relapse behaviors that cause psychological and physical dependence on a drug [83]. Specifically, common drug addictions include opium addiction, heroin addiction, methamphetamine addiction, marijuana addiction, and cocaine addiction [84], and so on. In recent years, a large number of SMILES strings have been used to train DL models due to the high parallelism capability and training efficiency of transformer models [85]. Thus, the transformer provides a more efficient option for drug addiction solutions. Next, we will specifically explore some applications of transformers in drug addiction in the field of drug discovery.

Due to the complexity of the mechanisms of drug addiction, the design of antiaddiction drugs remains a major challenge. In 2023, to address this challenge, Zhu et al. [18] constructed topological inferential drug addiction learning (TIDAL), a framework that was built by incorporating a multi-scale topological Laplace operator, a deep bi-directional transformer, and ensemble-assisted neural networks (EANNs). Among them, the transformer was an self-supervised learning (SSL)-based DL model inspired by NLP. Then, they validated the state-of-the-art of the proposed TIDAL framework on multiple addiction-related data sets. Finally, through some experiments, TIDAL revealed some information about 12 existing anti-addiction drugs. The results demonstrated the strength of the proposed TIDAL framework for modeling and analyzing addiction data.

**Fig. 6:**
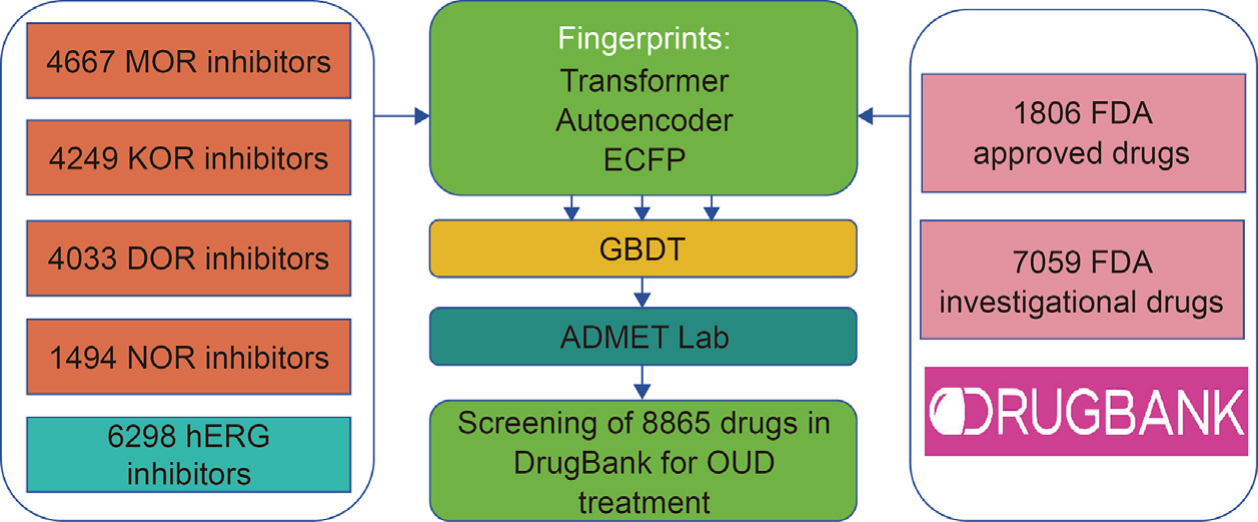
Schematic of the machine learning (ML) platform used to screen the DrugBank database for the treatment of opioid use disorder (OUD). In total, 8865 DrugBank drugs were screened for efficacy against major opioid receptors, the human ether-à-go-go-related gene (hERG) side effects, and the absorption, distribution, metabolism, excretion, and toxicity (ADMET) properties [19]. MOR: mu opioid receptor; KOR: kappa opioid receptor; DOR: delta opioid receptor; NOR: nociceptin opioid receptor; ECFP: extended connectivity fingerprints; GBDT: gradient boosting decision tree; FDA: Food and Drug Administration. Reprint from Ref. [19] with permission.

Opioid use disorder (OUD) is a chronic, relapsing disease that requires the urgent development of safer and more effective medications, with one valuable option being drug repurposing. Feng et al. [19] first summarized data sets of inhibitors for the four major opioid receptors in 2023. They then constructed ML models. A schematic of their ML platform is shown in Fig. 6 [19]. Inspired by NLP, they generated two molecular fingerprints via transformer and autoencoder models, respectively, along with a traditional 2D fingerprint called extended-connectivity fingerprints (ECFP). With these ML models, they carefully evaluated the binding affinity of DrugBank drugs at different binding thresholds. Further, they also performed a detailed analysis of the absorption, distribution, metabolism, excretion, and toxicity (ADMET) prediction results, providing a very meaningful tool for the treatment of OUD.

The psychosocial disorder caused by people's long-term use of cocaine is called cocaine addiction, and it has caused many deaths worldwide [86]. With the introduction of ChatGPT, a transformer-based chatbot, a new era of AI has arrived. Recently, Wang et al. [11] have fully utilized ChatGPT to facilitate the development of multi-targeted anti-cocaine addiction drugs. Additionally, they equipped GPT-4 with multiple plugins to make it more powerful. Generating optimal drug-like molecules with desired properties was the main goal of this study; therefore, they mathematically and statistically enhanced the generative network complex (GNC) model with the relevant suggestions provided by ChatGPT to obtain anticocaine addiction leads targeting multiple transporter proteins. Moreover, they further examined the binding affinity of multiple targets simultaneously. This study utilized the powerful function of GPT-4 in the design of anti-addiction drugs and introduced an innovative approach to the drug discovery process.

These examples show that transformers can be effectively applied at the bioinformatics level in the field of anti-drug addiction, providing new ideas for the design of anti-drug addiction medicines [87]. Specifically, it can improve the ability to characterize the structure and function of molecules through a self-attention mechanism. Moreover, it generates new molecules with properties such as antiviral activity, toxicity, and solubility through multi-objective optimization and property control [81]. However, its positional encoding may affect the spatial structure and stereochemical representation of the molecules. With the popularity of LLMs nowadays, combining transformers with other AI techniques will be more widely used in the field of anti-addiction in the future [88]. At the same time, researchers need to pay attention to the ethical and safety issues in addiction treatment to ensure the feasibility and sustainability of the technology application.

### 3.7 Small data set challenges

In the field of drug discovery, limitations imposed by various conditioning factors often lead to inadequate sample sizes in certain data sets. Fortunately, various ML and DL algorithms, including support vector machines, random forests, and other techniques, can address this challenge [89]. Specifically, the transformer, as one of these algorithms, yields unexpected results on small data sets, leveraging pre-trained models [90]. Subsequently, the transformer combined transfer learning and data augmentation techniques to adapt to experiments with limited data [91]. Moreover, the transformer can be integrated with pertinent domain knowledge, enhancing its performance on small data sets. Therefore, considering the aforementioned conditions, the application areas of the transformer on small data sets encompass drug discovery, image analysis, bioinformatics, the financial field [92], and more. We introduce the application of transformer to small data sets in drug discovery.

Hayes et al. [22] employed three graph-based models to predict target attributes from small data sets. Notably, all three models are grounded in the Merriman-Bence-Osher (MBO) framework, with one of them integrating the transformer architecture. The research was subsequently applied to the Ame, Bace, BBBP, Beet, and ClinTox data sets, respectively. It is important to mention that only about 1% of the data within these five data sets was labeled. Remarkably, the outcomes were unforeseen, as the model proposed in this paper yielded favorable results even with the limited labeled data. In particular, when these models were juxtaposed with others using the area under the receiver-operating characteristic curve (ROC-AUC) metric, they exhibited superior performance. Furthermore, the authors also conducted additional experiments to increase the proportion of labeled data to 2% and 5%. At these increments, it was observed that the model's outcomes were still elevated. Lastly, the paper delves into residue and similarity (R-S) scores and R-S indices on benchmark data to provide an interpretation of the models. In summary, the three MBO-based methodologies proved to be potent tools in scenarios where labeled data is in short supply.

**Fig. 7:**
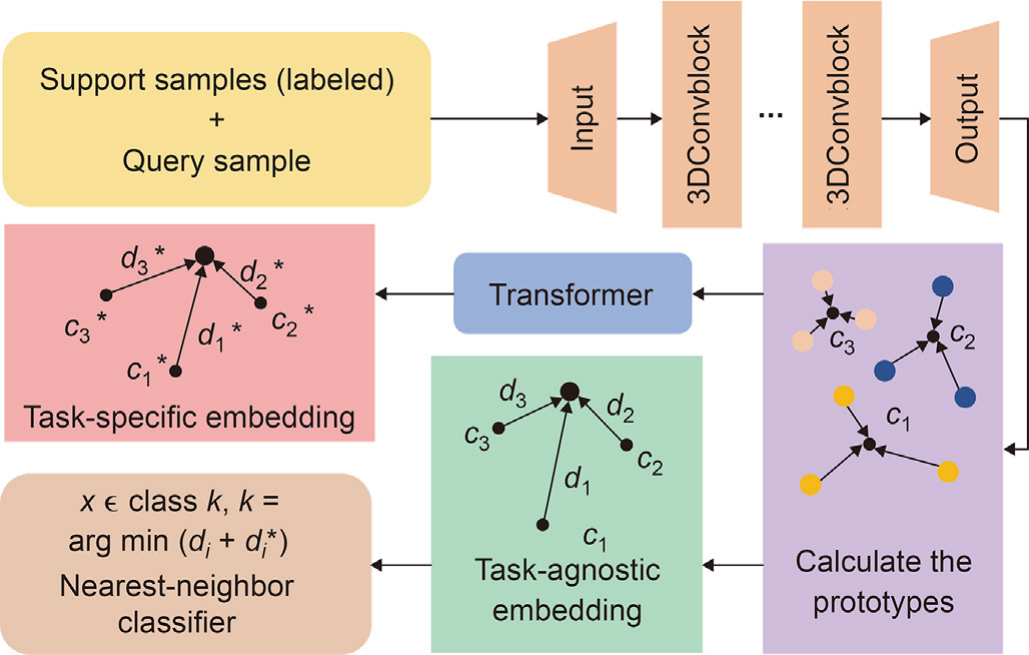
Flowchart of the few-shot learning approach for subtomogram classification. Suppose there was a data set with support samples that contained three classes and labeled samples for each class, these samples were first processed with a three-dimension (3D) encoder and this process placed the samples into an embedding space. Then the prototypes of each class were computed and divided into two routes. One of them generated a task-specific embedding space through the transformer, and the other one generated a task-agnostic embedding. The query sample x was simultaneously projected into both embedding domains, where the distances from x to the prototypes within each domain were computed. These distances were then aggregated to serve as the basis for classification through a nearest-neighbor classifier [23]. ∗ represents the prototype of different class and the distances between sample and prototype are calculated in a task-specific embedding space. *c*_1_, *c*_2_, or *c*_3_ means a prototype for class one, two, or three, and *d*_1_, *d*_2_, or *d*_3_ means the distance between the query sample and protoype *c*_1_, *c*_2_, or *c*_3_. Reprint from Ref. [23] with permission.

Li et al. [23] discussed the challenges in cryo-electron tomography (cryo-ET) for recognizing and recovering macro-molecular structures in cellular environments. Generally, despite advances in automation for subtomogram collection, obtaining structural labels remains computationally and labor-intensive. Hence, the proposed solution introduced a few-shot learning approach for subtomogram classification, allowing classification of unseen structures with minimal labeled samples. The schematic diagram of this scheme is shown in Fig. 7 [23]. Experimental results on simulated and real data sets demonstrated competitive accuracy, surpassing baseline methods. Specifically, the approach performed well with as few as one labeled sample, achieving an accuracy of 0.7644. In short, the method showed significant improvement over existing techniques, excelling in generalization to various cellular components, highlighting its potential in advancing structural biology *in situ*.

Torres et al. [93] considered the significance of predicting chemical toxicity and adverse side effects in drug discovery. Specifically, the proposed approach, few-shot graph neural network (GNN)-transformer (FS-GNNCvTR), addressed the challenge of low-data toxicity and side effect prediction. Meanwhile, they highlighted the role of GNNs and transformer networks in enhancing molecular property prediction (MPP). The method combined a convolutional transformer to model local spatial context in molecular graph embeddings with a two-module meta-learning framework for iterative parameter updating across few-shot tasks. As a result, experimental results on Tox21 and SIDER data sets demonstrated the superior performance of FS-GNNCvTR compared to standard graph-based methods, underscoring its potential in advancing drug development.

In drug discovery, the advantage of using transformers on small molecule data sets lies in their ability to generalize, making them effective in feature extraction [94]. This capability helps in learning effective representations from small molecule data sets and also makes transformers resistant to overfitting [95]. However, challenges remain due to the limited size and diversity of these data sets [64], which can hinder the transformers’ ability to learn sufficiently generalized representations. Additionally, interpretability of transformers on small data sets is a significant challenge [96].

Future advancements could involve developing specialized attention mechanisms tailored for small molecule data sets or proposing specialized small-sample learning algorithms [97]. These approaches aim to maximize the use of limited training data and improve model performance in drug discovery applications.

### 3.8 Chemical and biological image analysis

Following the proposal of the transformer model, researchers quickly explored its applicability in chemical/biological image analysis, focusing initially on molecular structure recognition, MPP [98], and omics data analysis. The introduction of the vision transformer (ViT) in 2020 brought new perspectives to chemical/biological image analysis [99]. Subsequently, numerous studies utilized transformer models to predict molecular structures in chemical reactions, optimizing reaction conditions and enhancing yields [100]. Furthermore, transformers have opened new avenues in drug discovery by screening potential drug molecules, identifying drug targets, and optimizing drug structures [101]. As transformer applications in chemical image analysis deepen, researchers have explored more efficient model structures, feature extraction methods, and computational frameworks [102].

Khokhlov et al. [25] introduced a transformer-based model for translating organic structure images into molecular structures, achieving notable accuracy. Their approach involved a generator performing simulations on molecular training data collected from the PubChem database. A probability-based screening method identified rare or complex molecules, followed by rendering augmentations and model training. The transformer-based architecture demonstrated a significant advantage in recognizing optical images of chemical structures, achieving 90.7% accuracy on the selected data set.

**Fig. 8:**
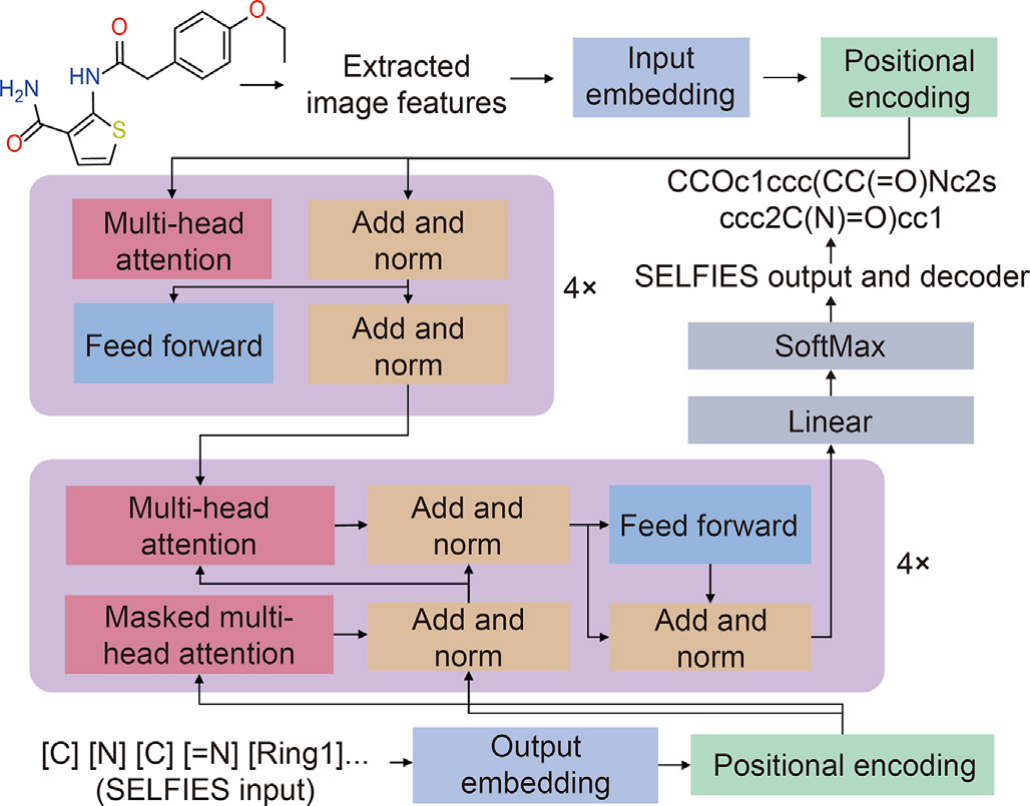
Process diagram of training the transformer model in deep learning (DL) for chemical image recognition (DECIMER). It consists of four encoder-decoder layers and eight parallel attention heads. In detail, the dimension size of the attention is 512 and the dimension size of the feedforward network is 2048. The model extracted the images into vector format as features. The loss was computed through sparse categorical cross-entropy, which compared the actual self-referencing embedded strings (SELFIES) with the predicted ones [26]. Reprint from Ref. [26] with permission.

Rajan et al. presented the DL for chemical image recognition (DECIMER) project, which leveraged transformer-based models for unraveling molecular structures from images, and its flowchart is depicted in Fig. 8 [26]. By employing a transformer-based framework and EfficientNet-B3 for image feature extraction, the project achieved a remarkable 96% accuracy in predicting SMILES strings. This innovative method streamlined the image-to-SMILES conversion process and marked a significant milestone in computational chemistry.

Yoo et al. [103] introduced a transformer-based approach for image-to-graph conversion, revolutionizing molecular structure recognition. Their model, incorporating ResNet and transformer layers for image encoding, exhibited outstanding accuracy in decoding chemical graphs. Trained on diverse data sets from PubChem and United States Patent and Trademark Office (USPTO), the model surpassed existing tools, showcasing advancements in molecular recognition.

Chemical images often feature intricate structures, and the transformer's attention mechanism excels at capturing long-range dependencies within these images [104]. Additionally, the transformer's ability to leverage transfer learning facilitates data set expansion, although challenges arise with images exhibiting high variance [105]. Despite these limitations, the transformer's application in chemical imaging may extend to various CV tasks, offering new possibilities in image classification, object detection, and image segmentation [106,107].

### 3.9 Chemical language understanding

Chemical language understanding is a critical area within the field of NLP. Initially, prior to the advent of the transformer-based models, chemical language understanding predominantly combined RNNs, as well as their variants such as long short-term memory (LSTM) networks and gated recurrent units (GRUs). However, these models struggled when dealing with lengthy sequences, failing to effectively capture long-range dependencies within the data. Fortunately, the introduction of the transformer's self-attention mechanism revamped sequence modeling in chemical language understanding [108]. Then, as transformers and their variants continued to evolve, these models were pre-trained by processing extensive corpora of chemistry-related texts. As a result, this enabled the models to encapsulate the linguistic patterns and knowledge inherent to the domain of chemistry, facilitating high-quality chemical language understanding [109]. Consequently, the transformer-based models are leveraged to tackle various tasks in chemical text processing, including chemical reaction analysis [5], drug discovery [110], and chemical patents analysis [111]. These advancements have been instrumental in propelling the development of chemical language understanding, and the relevant studies are described in more detail below.

**Fig. 9:**
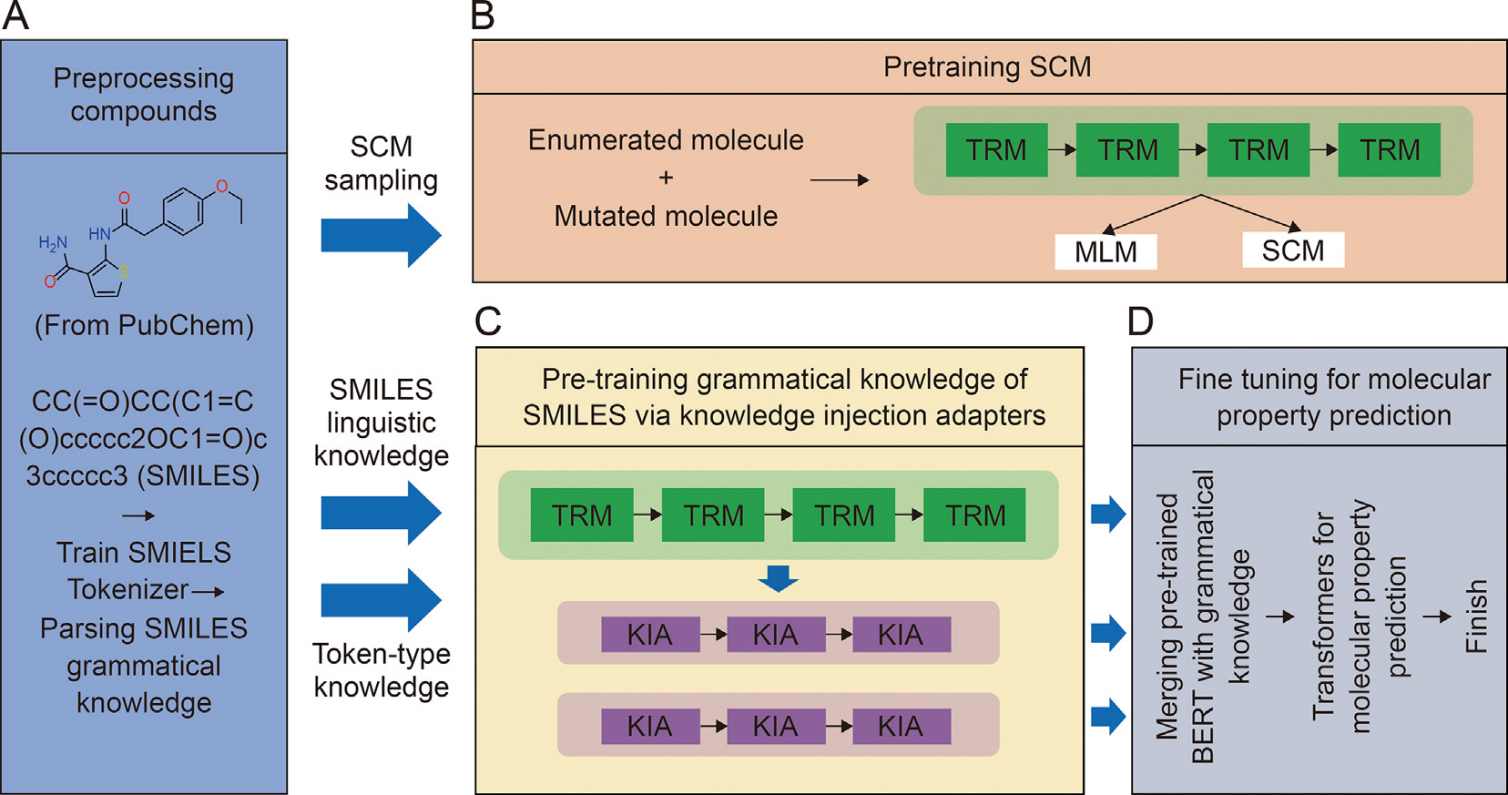
Schematic illustration of the overall model with injected grammatical knowledge. (A) Preprocess simplified molecular-input line-entry system (SMILES). (B) Pretrain bidirectional encoder representations from transformers (BERT). (C) Use K-adapters to pretrain the parsed knowledge to enhance the understanding of SMILES in pre-trained BERT. (D) Predict molecular properties using pre-trained BERT and grammatical knowledge. Afterwards, there are two steps for tokenization processing. The syntax structure of SMILES symbols is analyzed through their connectivity and type [27]. SCM: same compound model; TRM: transformer; MLM: masked language model; KIA: knowledge injection adapter. Reprint from Ref. [27] with permission.

Lee and Nam employed a training strategy termed the “same compound model” to enhance the understanding of SMILES grammar, as depicted in Fig. 9 [27]. They further pre-trained a BERT model to label SMILES representations. In this study, they utilized the K-adapter technique, which enabled the injection of SMILES information into the BERT model, thereby augmenting its proficiency in comprehending SMILES notation. Lee and Nam’s novel approach of injecting SMILES representations into the BERT model proved to be an effective strategy for understanding chemical languages. This methodology holds promise for extension to other chemical languages, potentially advancing the comprehension and analysis of diverse chemical notations.

Born and Manica [28] introduced the Regression Transformer (RT) model, capable of processing both textual and numerical data for sequence modeling. The RT was subsequently employed to tackle regression tasks, even in scenarios where previous operations incurred losses. The findings, demonstrated on two reaction data sets, revealed that the RT exhibited promising capabilities. It not only predicted reaction yields with comparable accuracy to traditional transformers but also demonstrated the potential to substitute specific precursors, leading to the discovery of novel reactions with higher predicted yields than the seed reaction. This underscores the RT's potential to advance the field of chemical synthesis and reaction prediction.

Morris et al. [112] trained a transformer-based model for translation between text-based representations of molecules. Specifically, they first collected and modeled the transformation of the SMILES and International Union of Pure and Applied Chemistry (IUPAC) chemical names of a large number of molecules. Therefore, they collected three data sets, the human immunodeficiency virus type 1 (HIV-1) Protease, AID 652067, and AID 1053197 data sets. Later, this study converts SMILES to IUPAC with the help of transformer's encoder-decoder architecture. The final experiment was performed on 83,000,000 molecules and molecular affinity predictions were made. In summary, this experiment chose AUC as the evaluation metric in performing molecular binding affinity prediction, and the results showed that the data-driven model based on trained translation embeddings demonstrated higher AUC than using untrained embeddings on multiple binding affinity prediction tasks.

In the field of chemical language understanding, transformers are expert in capturing complex patterns and dependencies within molecular text representations during pre-training [113]. Accordingly, this capability enhances the organization of chemical properties in latent embeddings, deeply contributing to understanding of molecular structures [114]. Meanwhile, the model's capacity to distinguish relationships between different chemical element positions is a powerful tool for predicting binding affinities [115]. However, training large-scale transformer models requires high computational costs and risks overfitting problems [116]. Additionally, interpreting the complex inner workings of the transformer model can be challenging [90]. In the future, the promising trend in chemical language understanding involves leveraging visualization techniques to illustrate the discriminatory abilities of the transformer model in embedding space [117]. Correspondingly, this visual insight into molecular relationships provides a valuable resource for researchers, ensuring that the transformative potential of transformers is fully realized in deciphering the language of chemistry [118].

### 3.10 Single cell data

Single-cell analysis is an all-encompassing and in-depth study of a single cell, which can reveal the heterogeneity and function of the cell and how the cell functions in its microenvironment [119]. To realize single-cell analysis, researchers have adopted various methods, such as single-cell sequencing [120], mass spectrometry [121], *in situ* hybridization [122], and others, which can obtain information about a cell's genome, transcriptome, proteome, metabolome, and cell-cell interactions [123]. However, single-cell research also faces some challenges, such as how to effectively parse and understand large amounts of high-dimensional data, which requires powerful computational capabilities and advanced algorithms [124,125]. In recent years, the transformer has attracted the attention of researchers, and it can be successfully applied to single-cell research for data mining and knowledge discovery at the bioinformatics level [126]. Next, we will discuss the application of the transformer model in single cells in detail and look forward to its future development trend.

**Fig. 10:**
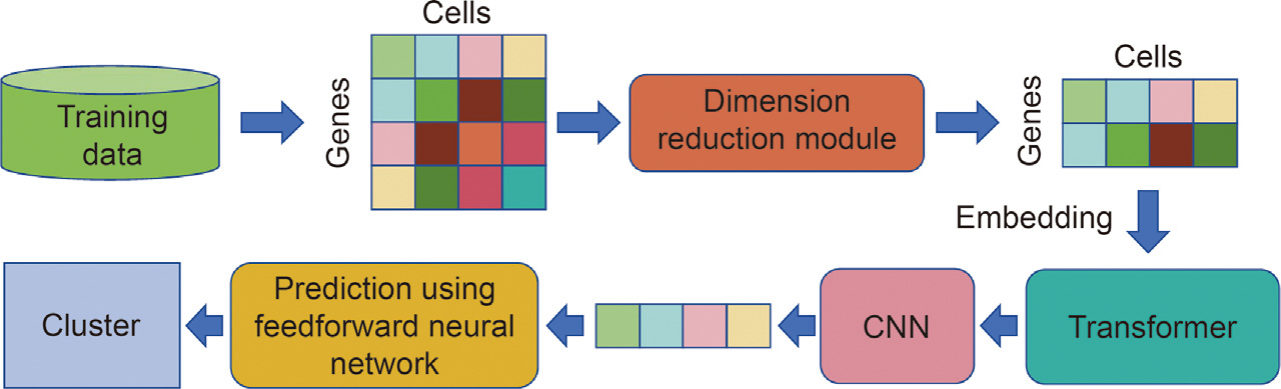
Structure of TransCluster. The inputs to TransCluster are the gene expression matrix of the cell and the category of the cell [127]. CNN: convolutional neural network. Reprint from Ref. [127] with permission.

To address the major challenge of accurate cellular annotation of single-cell transcriptional data, Song et al. [127] proposed a novel cell-type recognition tool called TransCluster in 2022. It was based on the transformer framework and was suitable for the analysis of single-cell transcriptome maps, and Fig. 10 [127] shows the structure of the TransCluster. First, it utilized the dimensionality reduction method of linear discriminant analysis (LDA) to preprocess the gene expression matrix to improve the representativeness and differentiation of the features. Second, it used a transformer-based DL model to learn features from the dimensionality-reduced data. Finally, experimental results on cellular data sets from several different human tissues showed that TransCluster had high accuracy and robustness. Notably, this is the first time that the transformer model has been applied to the field of single-cell category prediction.

To identify and characterize the cellular composition of complex tissues, single-cell RNA sequencing (scRNA-seq) is a very useful technique [128]. Jiao et al. [129] developed a deep neural network (DNN) model entitled scTransSort in 2023, which was based on transformer pre-training and can intelligently extract cellular features and predict cell types using a self-attention mechanism. Meanwhile, to address the sparsity and computational complexity of scRNA-seq data, scTransSort adopted an innovative approach to convert gene expression data into gene expression embedded blocks, which improved the efficiency and accuracy of cell type identification. Furthermore, scTransSort demonstrated excellent performance in cellular experiments on 35 human and 26 mouse tissues and also had strong robustness and generalization capabilities.

The development and reproducibility of single-cell research rely on the consistent transfer of annotations from a reference data set to a query data set [130]. To achieve this goal, Chen et al. [131] introduced a DL model called transformer for one-stop interpretably cell-type annotation (TOSICA) in 2023, which was based on the transformer's multi-head self-attention mechanism and was capable of performing interpretable cell-type annotations using biologically understandable entities such as pathways or regulators. They applied TOSICA to scRNA-seq data from tumor-infiltrating immune cells and CD14^+^ monocytes in coronavirus disease 2019 (COVID-19), demonstrating the advantages of TOSICA in revealing rare cell types, heterogeneity, and dynamic trajectories associated with disease progression and severity. As an innovative model that applies transformer architecture to the analysis of single-cell histology data, TOSICA provided an effective and interpretable opportunity for cell-type annotation across large-scale data sets.

In addition to the ones mentioned above, transformers have many applications in single-cell analysis [132,133]. For example, De Waele et al. [134] proposed a cytosine-phosphate-guanine (CpG) transformer, a method for single-cell methylation interpolation that achieves state-of-the-art interpolation performance with limited time and computational budget. Additionally, a single-cell bi-directional encoder representation from the transformer (scBERT) was developed by Yang et al. [135] for cell-type annotation of scRNA-seq data. Moreover, CIForm, a transformer-based supervised method for cell-type annotation of large-scale scRNA-seq data, was introduced by Xu et al. [136]. Specifically, one advantage of a transformer in single-cell data is the ability to capture remote dependencies from a global perspective. Another advantage is the ability to explain biological phenomena through the attention mechanism in the transformer [137]. However, the transformer also faces some challenges, such as high computational complexity and difficulty handling data sparsity. In conclusion, the application of a transformer at the bioinformatics level in single-cell analysis provides us with a new perspective and tool to explore the mystery and complexity of single cells.

## 4 Perspectives

In this review, we delineate the objectives and methodology for a comprehensive investigation into the algorithmic and practical applications of transformer-based ML techniques in drug discovery, chemistry, and biology. We have specifically discussed discovery aspects such as transformer-assisted protein engineering, biomolecular dynamics, drug target identification, and transformer-enabled drug VS. Transformer applications to drug lead optimization, drug addiction, small data set challenge, chemical and biological image analysis, chemical language understanding, and single cell data are reviewed.

The findings of this review hold promise for significantly advancing our understanding of complex molecular systems and expediting discoveries in drug discovery, chemistry, and biology.

### 4.1 The role of LLMs in drug discovery

LLMs, like ChatGPT, are revolutionizing drug discovery by significantly enhancing data mining, hypothesis generation, molecular design, predictive modeling, and clinical trial management. These models can swiftly scan and analyze vast amounts of scientific literature, patents, and clinical trial data to identify patterns and relationships that may not be immediately apparent to human researchers. By generating new hypotheses regarding drug interactions and potential repurposing opportunities, LLMs accelerate the initial stages of drug discovery. Furthermore, their ability to assist in designing new drug molecules and optimizing molecular properties streamlines the design process, reducing the time and cost associated with traditional experimental approaches. Predictive models developed by LLMs can forecast PK, toxicity, and clinical outcomes, enhancing decision-making and candidate selection before costly clinical trials. In clinical trial design and management, LLMs identify appropriate patient populations, predict trial outcomes, and optimize trial protocols, leading to faster and more cost-effective trials with a higher likelihood of success.

Overall, the integration of LLMs and transformer-based models in drug discovery is poised to make the process more efficient, cost-effective, and data-driven. However, it is essential to use these models in conjunction with expert human judgment and experimental validation to confirm AI-generated insights. Addressing ethical and regulatory challenges associated with AI in drug discovery will be crucial for the successful implementation of these technologies, ensuring that the benefits of AI advancements are fully realized while maintaining safety and efficacy standards.

### 4.2 Multimodal learning for omics data

The analysis of omics data in drug discovery is fraught with challenges due to its intrinsic complexity and heterogeneity. Comprising various layers of biological information, including genomics, proteomics, transcriptomics, metabolomics, and other omics data sets, this multifaceted landscape demands sophisticated analytical methodologies. Transformer-based multimodal learning approaches have emerged as a promising solution to these challenges, offering a unified framework capable of integrating disparate data modalities seamlessly. This integration enables a holistic view of biological systems, unlocking synergistic insights that transcend individual data silos and facilitating comprehensive data fusion. Leveraging the inherent structure and relationships within these multimodal data sets, transformer models allow for a nuanced understanding of the complex chemical and biological phenomena that are crucial for drug discovery.

By synthesizing genomic, proteomic, transcriptomic, and metabolomic information, researchers can uncover intricate chemical and biological interactions, elucidate regulatory mechanisms, and identify biomarkers with unprecedented accuracy. Such insights are invaluable for drug development, where understanding MD and disease mechanisms is essential. Transformer models enhance the ability to identify novel therapeutic targets, predict drug efficacy, and optimize safety profiles, thus accelerating the entire drug discovery process. These models also aid in developing tailored treatment strategies, fostering advancements in personalized medicine by providing deeper insights into patient-specific responses to therapies.

Transformer-based multimodal learning approaches are transforming drug discovery and development. By integrating diverse omics data, these models enable researchers to generate more accurate and comprehensive insights, facilitating the discovery of novel drugs and the development of personalized treatment strategies. This advancement paves the way for transformative progress in healthcare and biotechnology, ultimately leading to more effective and individualized therapeutic interventions.

### 4.3 Catalyst and enzyme screening

Catalysts and enzymes play a crucial role in drug discovery by accelerating chemical and biochemical reactions, often leading to more efficient outcomes. Traditionally, the selection of these catalysts relied heavily on the expertise and experience of experimental personnel. However, with the advent of computational chemistry and advancements in drug discovery, this selection process has increasingly incorporated theoretical calculations. The transformer model has shown exceptional performance in this domain, revolutionizing the selection of catalysts in chemistry and drug discovery. The transformer's powerful self-attention mechanism and ability to process long sequence data enable it to capture complex relationships in chemical and biochemical reactions effectively. This capability allows for the efficient screening of catalysts and enzymes, enhancing both the efficiency and selectivity of chemical reactions. Such advancements are particularly significant in organic synthesis and energy conversion, where precise catalyst selection is crucial. By leveraging these capabilities, the transformer model can identify the most effective catalysts, thus accelerating the drug discovery process and improving the development of new therapeutics.

Furthermore, the transformer model's multi-task learning and transfer learning capabilities allow it to be applied flexibly across different chemical reactions, enzymes, and catalysts. This flexibility enhances research efficiency by enabling the model to adapt to various contexts within drug discovery. As a result, the transformer model not only optimizes catalyst selection but also streamlines the overall research process, paving the way for significant advancements in the development of new drugs and therapeutic strategies.

### 4.4 Customizing transformer models for drug discovery

In drug discovery, the adaptability of the transformer architecture allows for tailored solutions to unique challenges. Customized transformer models, refined on domain-specific data sets, excel in tasks like sequence classification, molecular generation, and structure prediction. By incorporating domain knowledge and employing transfer learning, these models offer superior accuracy and robustness.

Customized transformer models leverage domain expertise and ML techniques to discern intricate patterns in biological data. These models advance molecular recognition and design by expediting lead compound identification and optimizing molecular structures. In drug discovery, they facilitate predictive modeling and *de novo* compound generation. In systems biology, they elucidate biological networks and predict phenotypic traits, driving precision medicine and therapeutic interventions.

Overall, customized transformer models revolutionize drug research, enabling deeper insights and accelerating advancements in healthcare and biotechnology.

## 5 Conclusions

In this review, we provide a comprehensive overview of the applications of transformer-based models in drug discovery, as well as chemistry and biology. Specifically, we discuss primary areas, such as protein design and protein engineering, MD, drug target identification, transformer-enabled drug VS, drug lead optimization, drug addiction, small data set challenges, chemical and biological image analysis, chemical language understanding, and single cell data. As transformers continue to reshape the landscape of drug discovery, the integration of LLMs, multifunction transformers, and customized transformer models heralds a future where our comprehension of drug discovery is not only deep but also constantly evolving and flexible. This review underscores the promising outlook for these technologies and paves the way for a significant advancement in drug discovery. We believe that the transformer-based models will become a powerful tool for solving various difficult problems in the fields of drug discovery and other sciences.

## CRediT authorship contribution statement

**Jian Jiang:** Writing – review & editing, Writing – original draft, Methodology, Conceptualization. **Long Chen:** Writing – original draft, Data curation. **Lu Ke:** Writing – original draft, Data curation. **Bozheng Dou:** Data curation. **Chunhuan Zhang:** Writing – original draft, Data curation. **Hongsong Feng:** Data curation, Software. **Yueying Zhu:** Investigation, Data curation, Conceptualization. **Huahai Qiu:** Investigation, Data curation, Conceptualization. **Bengong Zhang:** Methodology, Funding acquisition, Conceptualization. **Guo-Wei Wei:** Writing – review & editing, Supervision, Methodology, Funding acquisition, Conceptualization.

## Declaration of competing interest

The authors declare that there are no conflicts of interest.
